# Determination of Phenolic Compounds in Various Propolis Samples Collected from an African and an Asian Region and Their Impact on Antioxidant and Antibacterial Activities

**DOI:** 10.3390/molecules26154589

**Published:** 2021-07-29

**Authors:** Soumaya Touzani, Hamada Imtara, Shankar Katekhaye, Hamza Mechchate, Hayat Ouassou, Ali S. Alqahtani, Omar M. Noman, Fahd A. Nasr, Hugo Fearnley, James Fearnley, Anant Paradkar, Ilham ElArabi, Badiaa Lyoussi

**Affiliations:** 1 Laboratory of Natural Substances, Pharmacology, Environment, Modeling, Health and Quality of Life (SNAMOPEQ), Faculty of Sciences Dhar El Mahraz, University Sidi Mohamed Ben Abdellah, P.O. Box 1796, Fez 30000, Morocco; Soumaya.touzani@usmba.ac.ma (S.T.); ilham.elarabi@usmba.ac.ma (I.E.); badiaa.lyoussi@usmba.ac.ma (B.L.); 2 Faculty of Arts and Sciences, Arab American University Palestine, Jenin P.O. Box 240, Palestine; 3 Centre for Pharmaceutical Engineering Science, University of Bradford, Bradford BD7 1DP, UK; S.Katekhaye@bradford.ac.uk; 4 Nature’s Laboratory Ltd., Unit 3b, Enterprise Way, Whitby, North Yorkshire YO22 4NH, UK; hugo.fearnley@beevitalpropolis.com (H.F.); james.fearnley@beevitalpropolis.com (J.F.); a.paradkar1@bradford.ac.uk (A.P.); 5 Laboratory of Inorganic Chemistry, Department of Chemistry, University of Helsinki, P.O. Box 55, FI-00014 Helsinki, Finland; hamza.mechchate@helsinki.fi; 6 Laboratory of Bioresources, Biotechnology, Ethnopharmacology and Health, Faculty of Sciences, Mohammed First University, Oujda 60000, Morocco; hayatouassou@gmail.com; 7 Department of Pharmacognosy, College of Pharmacy, King Saud University, Riyadh 11451, Saudi Arabia; alalqahtani@ksu.edu.sa (A.S.A.); onoman@ksu.edu.sa (O.M.N.); fnasr@ksu.edu.sa (F.A.N.)

**Keywords:** propolis, phenolic profile, HPLC, antioxidant and antibacterial activity

## Abstract

The biological activities of propolis samples are the result of many bioactive compounds present in the propolis. The aim of the present study was to determine the various chemical compounds of some selected propolis samples collected from Palestine and Morocco by the High-Performance Liquid Chromatography–Photodiode Array Detection (HPLC-PDA) method, as well as the antioxidant and antibacterial activities of this bee product. The chemical analysis of propolis samples by HPLC-PDA shows the cinnamic acid content in the Palestinian sample is higher compared to that in Moroccan propolis. The results of antioxidant activity demonstrated an important free radical scavenging activity (2,2-Diphenyl-1-picrylhydrazyl (DPPH); 2,2′-azino-bis 3-ethylbenzothiazoline-6-sulphonic acid (ABTS) and reducing power assays) with EC_50_ values ranging between 0.02 ± 0.001 and 0.14 ± 0.01 mg/mL. Additionally, all tested propolis samples possessed a moderate antibacterial activity against bacterial strains. Notably, Minimum Inhibitory Concentrations (MICs) values ranged from 0.31 to 2.50 mg/mL for Gram-negative bacterial strains and from 0.09 to 0.125 mg/mL for Gram-positive bacterial strains. The S2 sample from Morocco and the S4 sample from Palestine had the highest content of polyphenol level. Thus, the strong antioxidant and antibacterial properties were apparently due to the high total phenolic and flavone/flavonol contents in the samples. As a conclusion, the activities of propolis samples collected from both countries are similar, while the cinnamic acid in the Palestinian samples was more than that of the Moroccan samples.

## 1. Introduction

Generally, physiological and endogenous reactive oxygen species (ROS) are derivatives of oxygen and are generated during the mitochondrial respiratory chain as natural by-products of normal cell activity [1]. Oxidative stress refers to the imbalance between cellular antioxidant response and processes that generate reactive oxygen species [2]. Oxidative stress damages various cellular components such as nucleic acids, proteins, and lipids, which causes many diseases such as cancer, diabetes, atherosclerosis, neurodegeneration, Alzheimer’s disease, and aging [3,4]. However, several synthetic antioxidants have been proposed for the prevention and treatment of certain diseases, but their toxicity has led to harmful effects in their use [5,6].

On the other hand, according to the World Health Organization, antibiotic-resistant bacteria pose a serious threat to the world’s health, although antibiotics have played an effective role over the past century in fighting many diseases and infections [7,8]. There is a growing effort in the search for natural compounds displaying biological activities that could be efficiently harnessed for managing diseases and combating drug resistance. Natural products, including bee products, are used for therapeutic purposes as alternative drugs [9,10].

Propolis is a natural resinous mixture produced by honeybees (*Apis mellifera* L.) from the buds, leaves, bark, and exudates of plants [11,12]. Nowadays, propolis has become a natural alternative to drugs, and it is application is referred to as complementary or alternative medicine. Several studies have demonstrated the pharmacological activities of propolis such as analgesic–anesthetic activity [13], antifungal [14], anti-inflammatory, immunomodulatory activity [15], hepatoprotective [16], antidiabetic [17], and hypoglycemic and antihypertensive effects [18]. A variety of secondary metabolites contained in propolis such as phenolic acids, flavonoids, and volatile compounds are responsible for these biological activities [19,20].

The chemical composition of propolis is variable depending on the biogeographical zone origin, ecological habitat, climatic conditions, or even the season, as well as the production methods, processing, and storage conditions [21,22].

The International Honey Commission and many researchers from Japan, Korea, China, Russian, and Taiwan develop their own quality standards for propolis, which depend mainly on physicochemical properties and antioxidant content. The developed standard methods to evaluate the quality of propolis should be fast, low cost, accurate, reliable, and reproducible [23,24]. The aim of this study was, therefore, to identify phenolic compounds present in the samples using HPLC-PDA, which can be used as an indicator for the quality of propolis for two countries. The second objective was to determine the physicochemical parameters and biological properties of propolis samples collected from different geographic origins (Palestine and Morocco). The entire datasets were used to study the correlations between the evaluated parameters and to run the principal component analysis (PCA) for the discrimination of propolis samples.

## 2. Results and Discussion

### 2.1. Physicochemical Characterization of Propolis Samples

Propolis consists of 50% resin, 30% wax, and others components [25]. The physicochemical characterization results of propolis samples are illustrated in the Table 1. The highest wax level was found in sample P3 from Morocco with a value of 43.12%, while sample P4 from Palestine presented the lowest value (13.39%). The other propolis samples P5 and P2 contained 45.25% and 59.01% of resin component. The results of this work are in agreement with the limit elaborated by the Brazilian legislation [26]. In addition, the content of wax and resin found in Moroccan and Palestinian propolis is similar to the results obtained for Italian propolis, in which resin values ranged from 39.1% and 72.7%, while wax values ranged from 12.8% and 41.0% [25]. Touzani et al. [27] reported that resin and wax contents of Moroccan propolis sample were 59.01% and 20.31%, respectively. Regarding balsam content, the maximum content was found in sample P4 from Palestine with a value of 1.3%. This value was two folds higher than the minimum value seen in sample P5 coming from the same country with a value of 0.69% (Table 1). Moreover, others parameters indicate the quality of propolis such as the moisture and high water content [28]. As results, the moisture content values in P2 and P5 samples were 1.01% ± 0.01% and 2.79% ± 0.09%, respectively. These results showed the conformity with the standard limit established by the Brazilian legislation (not more than 8%) [23].

The pH value of all analyzed propolis samples varies between 4.2 to 5.2. The pH results were similar to those of others studies [8,26,29]. Moreover, an ash content value of 1.76% was observed in the P5 sample, while sample P4 showed an ash value of 5.11%. The ash content could be used as an indicator of adulterated propolis samples [28,30]. Thus, according to our results, all analyzed parameters showed conformity with the limit established by Brazilian legislation except for sample P4, in which the ash content was more than 5% [23].

### 2.2. HPLC Analysis

#### 2.2.1. Method Development and Calibration Curves

The HPLC analysis method was developed by referring to the earlier method reported by Watson et al. [31]. It was found that there was a linear regression of standards with relation to the ratio of the intensity (AU) of the analytic to the concentration (Table 2). The calibration curve was linear, with the determination coefficient (R^2^) value in the range of 0.9651–0.9999. High correlation coefficients and wide linear ranges of the investigated concentration ranges were observed for all the standards.

#### 2.2.2. Method Validation

To avoid interference in the analysis due to the PDA detector, we analyzed all the standards for their absorption maxima and UV spectra. The wavelength of 290 nm for simultaneous analysis was selected as the most suitable wavelength.

Considering the complexity in propolis composition and the detected peaks, the precision is reasonable. In the stability and repeatability studies, the % RSD of compounds displayed a range from 0.7 to 1.5 and from 0.5 to 1.9, respectively (Table 2). Results showed that the standards were stable at room temperature for 24 h, and the developed method was sufficiently effective for the routine analysis of propolis.

In the study, the lowest concentration at which an analyte can be detected (LOD) or quantified (LOQ) with acceptable precision and accuracy was calculated from the standard deviation of the response and the slope obtained from linear regression of the calibration curve. LOD and LOQ values reported as µg/mL (*n* = 3) are shown in Table 2. To confirm these values, standard solutions in the LOQ and LOD were prepared and submitted to HPLC analysis.

Accuracy was tested by the addition of standard compounds in the propolis sample solution, at three different concentration levels, 80%, 100%, or 120% of the sample concentration. The mean percentage recovery and mean RSD at these three different concentration levels of standards in propolis sample is described in Table 2.

#### 2.2.3. Estimation of Marker Compounds

**Figure 1:**
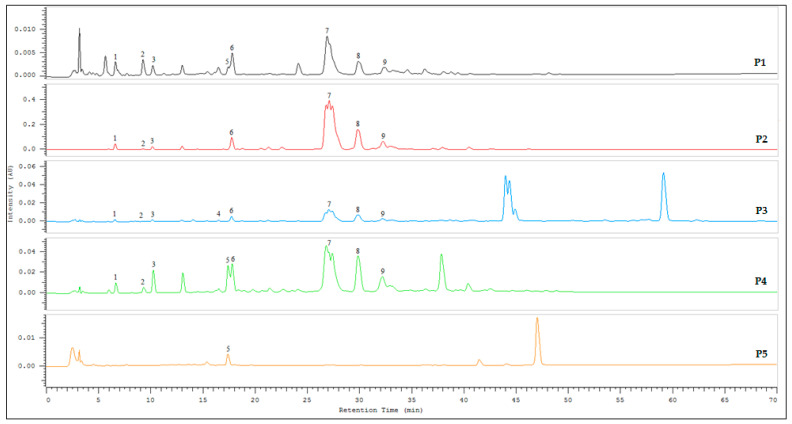
Chromatograms for propolis samples (P1, P2, P3, P4, and P5) with identified marker compound. 1. Caffeic acid, 2. *p*-coumaric acid, 3. ferulic acid, 4. quercetin, 5. cinnamic acid, 6. naringenin, 7. pinocembrine, 8. chrysin, and 9. galangin.

The quantified amounts of individual standards (µg/g) in the propolis samples obtained from Morocco and Palestine are as shown in Table 3. Amongst the tested standards, markers such as gallic acid, chlorogenic acid, rutin, and caffeic acid phenethyl ester (CAPE) were either not detectable or absent in the propolis samples. The variation in the propolis samples composition is dependent on the type of local flora at the site of collection [11,32]. The analysis suggest that sample P2 from Morocco and P4 from Palestine are good in quality considering the content of detected markers and the abundance of peaks in the chromatogram compared to other samples from respective countries (Figure 1).

Pinocembrine was observed to be a prominent component in the analyzed samples. The cinnamic acid content level in Palestinian samples was higher as compared to that in Moroccan propolis. Cinnamic acid is an organic acid that has low toxicity and has antioxidant and antibacterial activities [33]. The content of pinocembrine varied from 8721 (P1) to 352,001 (P2) µg/g. The chrysin content varied from 542 (P1) to 30,061 (P2) µg/g, whereas the galangin content varied from 234 to 25,014 µg/g. In general, for all samples, the remaining compounds were classified in the following order: naringenin > *p*-coumaric acid > ferulic acid > caffeic acid > quercetin. On the other hand, the work of Touzani et al. revealed that pinocembrin (83.4 mg/g) was the main highest compound among the identified compounds [27]. The chemical profile of Palestinian propolis was similar to that of Moroccan propolis. Several studies revealed the presence of several compounds including rutin, quercetin, kaempferol, caffeic acid, ferulic acid, chlorogenic acid, galangin, naringenin, CAPE, *p*-coumaric acid, chrysin, pinocembrin, apigenin, cinnamic acid, cinnamyl caffeate, gallic acid, and aromatic acids [27,32,33]. Our HPLC analysis results are in line with these studies. It is noteworthy that the phenolic acid and flavonoid compounds have been pinpointed as responsible for the antibacterial and the antioxidant properties [34,35]. Furthermore, as shown in the Figure 1, there are many compounds that have not been identified as a result of the huge and different amounts of compounds within the propolis samples.

### 2.3. Bioactive Compounds and Antioxidant Activity

Phenolic compounds are mainly present in propolis as flavonoids. The biological activities of propolis such as antioxidant and antimicrobial activities depend upon its phenolic compounds [10,36]. Several studies have showed that the variations in the chemical composition of the propolis are influenced by the type, origin, the raw material, and the extraction method [29]. The results of total phenolic, flavone/flavonol contents, and antioxidant capacity of different samples of propolis are shown in Table 4. Regarding propolis’ total phenolic content, the Palestinian propolis (P5) presented the lowest phenolic content of 74.71 ± 0.89 mg GAE/g, while the Moroccan propolis (P2) presented the highest amount of phenols (148 ± 1.31 mg GAE/g). The results of flavone and flavonol content were similar to those for total phenolic content, the highest total phenolic value was found in sample P2 with a value of 118 ± 1.92 mg QE/g of propolis, while a lower amount was observed in sample P5 with a value of 26.97 ± 2.44 mg QE/g of propolis. Results similar to ours were obtained in other studies [29,37]. The phenol content correlated positively with the flavone and flavonol contents (r = 0.953). Moreover, the total antioxidant capacity (TAC) varied from 48.01 ± 0.51 to 90.87 ± 2.91 mg AAE/g.

The antioxidant activities of the selected propolis samples were presented as EC_50_ values shown in Table 4. Propolis samples showed a stronger scavenging activity against DPPH radical with EC_50_ between 0.14 ± 0.01 and 0.02 ± 0.01 mg/mL. Furthermore, negative correlations were obtained between the antioxidant activity against DPPH radical and total polyphenol (r = −0.984), total flavones/flavonols (r = −0.978), and TAC activity (r = −0.915). This correlation agrees with many studies [38,39,40]. Antioxidant activity was also assessed by the ABTS assay. The results found that sample P2 from Morocco had the best activity with an EC_50_ value of 0.05 ± 0.001 mg/mL and sample P5 from Palestine presented the lowest EC_50_ of 0.43 ± 0.01 mg/mL, being the less active sample. In this section, no correlation was observed between phenolic compounds and antioxidant activity by ABTS unlike the results of DPPH. It should be noted that this result is different to previous results obtained by other authors [38,39].

Regarding the reducing power, the results illustrated in Table 4 showed that sample P2 had the most reducing activity (0.039 ± 0.001), while sample P5 had the lowest activity (0.094 ± 0.003). A possible effect of total phenolic content, flavone and flavonol content, and TAC could be seen through the resultant negative correlation with the EC_50_ of samples. The r values were r = −0.994, r = −0.914, and r = −0.984 respectively. Based on the results obtained in this section, we suspect that the antioxidant activity of selected propolis samples could be affected by the geographical areas as well as the total polyphenol and flavonoid contents [41].

### 2.4. Antibacterial Activity of the Propolis Sample

The antibacterial activity of propolis is one of the most documented pharmacological effects in the literature [42,43]. One of the mechanisms that have been demonstrated is that propolis affects the membrane permeability of microorganisms by disruption membrane potentials and adenosine triphosphate (ATP) production, while also decreasing bacterial mobility [42,44,45]. The agar diffusion method is a preliminary method used to test the ability of samples to inhibit bacterial growth [46], the results of propolis sample using this method are shown in Table 5. Against strains studied, sample P2 had the highest antimicrobial activity as the diameter of the zone of inhibition for *S. faecalis* (32.5 ± 1.02 mm) and *S. aureus* (31.2 ± 1.73 mm). Notably, P4 recorded the stronger antibacterial effect against *E. coli* 57 (21.8 ± 0.35 mm) and *P. aeruginosa* (20.33 ± 0.57 mm), while the lowest activities were observed on all strains for samples P3 and P5.

The highest resistance in *E. coli* 57 exhibited against cefuroxime, amoxicillin, cefotaxime, cephalothin, trimethoprim-sulfamethoxazole and ciprofloxacin, while *P. aeruginosa* exhibited resistance against trimethoprim-sulfamethoxazole and amoxicillin/clavulanate (Table 6).

The results seen in this study showed that Gram-positive bacteria are more sensitive than Gram-negative ones. These antimicrobial activity results are consistent with other experiments conducted on antibacterial activity of propolis [42,44,45]. In Table 7, *p*-coumaric acid was positively correlated with the zone of inhibition of selected propolis against *E. coli* 57 (r = 0.921), *E. coli* 97 (r = −0.968), and *P. aeruginosa* (r = 0.898), while ferulic acid positively correlated with the zone of inhibition of propolis against *E. coli* 97 (r = 0.947).

The MIC and MBC values of propolis samples are shown in Table 5. The MICs of tested bacteria was between 0.09 to 0.125 mg/mL. The Moroccan propolis, P2, exhibited an interesting bacterial effect against Gram-positive bacteria followed by the Palestinian propolis, P4. The sample exhibited moderate efficacy against Gram-negative microorganisms with an MIC between 0.31 to 2.50 mg/mL. Likewise, the MBCs values were closely similar for slightly higher propolis doses. Hence, the antibacterial effect of propolis could be related to the cell wall composition as well as the membrane structure of the test organism. Furthermore, a possible synergistic effect of the bioactive components such as polyphenols and flavonoids may be considered to be one of the main antibacterial agents [47,48,49,50]. Unlike the correlation in disc diffusion, the *p*-coumaric acid exhibited negative correlation with MIC values of propolis on *E. coli* (r = −0.978), *S. faecalis* (r = −0.915), and *S. aureus* (r = −0.935). However, only ferulic acid negatively correlated with MIC of propolis on *S. faecalis* (r = −0.992).

### 2.5. Multivariable Analysis

**Figure 2:**
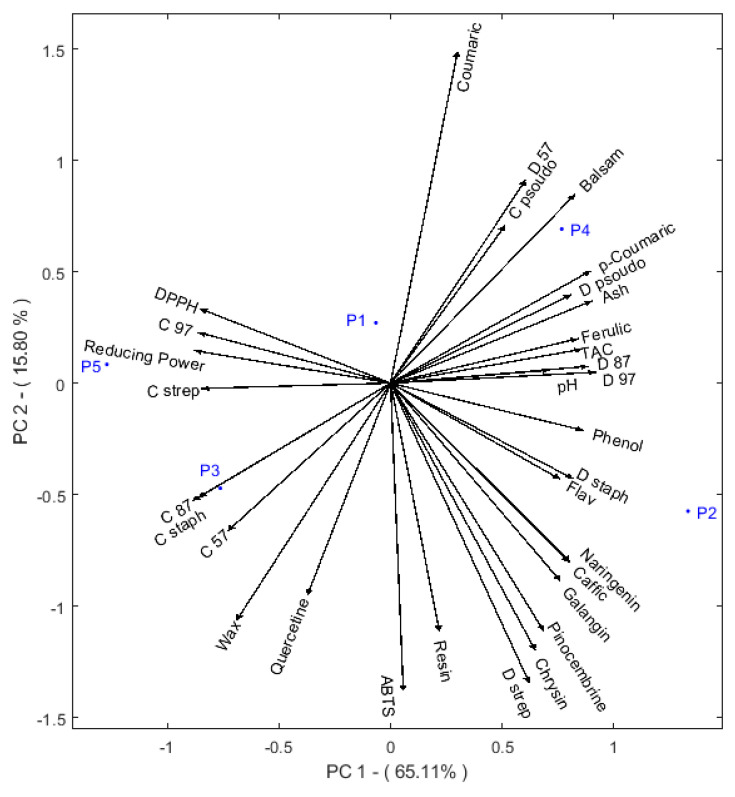
Principal component analysis (PCA) of the analyzed propolis samples using the assessed parameters as an input. Wax; resin; balsam; ash; pH; phenol: total phenolic; flav: flavone and flavonol; TAA: total antioxidant capacity; DPPH: 2,2-Diphenyl-1-picrylhydrazyl; ABTS: 2,2′-azino-bis (3-ethylbenzothiazoline-6-sulphonic acid; reducing power; caffeic: caffeic acid; coumaric: *p*-coumaric acid; ferulic: ferulic acid; quercetin; coumaric: cinnamic acid; naringenin; pinocembrin; chrysin; galangin; D: diameters of the inhibition zones; C: minimum inhibitory concentration; 87: *Escherichia coli* BLSE (ATB:87); 57: *Escherichia coli* (ATB:57); 97: *Escherichia coli* (ATB:97); Staph: *Staphylococcus aureus*; pseudo: *Pseudomonas aeruginosa*; strep: *Streptococcus faecalis*.

Regarding the distribution of the propolis samples, based on the assessed parameters, principal component analysis (PCA) was used. The PCA is a good tool for information extraction from multivariate matrices, and it concentrates on only a few components [51]. The propolis samples plotted are in blue, and the parameters are illustrated as black arrows. The first two PCs accounted for 65.11% and 15.80%, respectively, of total variation in the original data (Figure 2). The first PC explained more variability and correlated positively with the total phenolic and flavone and flavonol contents. Consequently, the same PC correlated also the identified bioactive compounds, except for quercetin, and antibacterial activities (assessed by diffusion method). As a result, a negative correlation between the PC and antioxidant and antibacterial properties (MIC except ABTS) can be noted. This activity correlated negatively with the content of coumaric acid, making it the suggested compound responsible for the observed ability to scavenge ABTS radical cations.

Considering the similarities of the samples and the component (PC1) that correlated the bioactive content of propolis samples and their antioxidant activities, the samples could be divided into two groups. As expected from the correlation between the parameters, the first composed group of P2 and P4 were found to have the highest total phenolic content, which will induce stronger antioxidant and antibacterial activities. These samples are located in the positive part of the plot, while the P3 and P5 samples are located in the negative part of the plot and presented the opposite properties compared to the P2 and P4 samples.

## 3. Materials and Methods

### 3.1. Source of Propolis

Propolis (honeybee propolis) used in this study were purchased from apiculturists from Morocco and Palestine. Based on information collected from beekeepers, the samples were numbered P1–P5. Table 8 provided the details of the predominant vegetation in the region/country of collection. 

### 3.2. Physicochemical Characterization of Propolis

The determination of resin, wax, balsam, and ash contents in samples was carried out as recommended by Papotti et al. [25]. The pH of propolis samples was measured by a pH meter based on the technique designated previously by Dias et al. [52]. The AOAC procedure was used to evaluate the moisture content in propolis samples [53].

### 3.3. Antioxidant Activities of Propolis Samples

Three methods were used to determine the antioxidant activities of samples against free radicals. The DPPH method was reported by Brand-Williams et al. [54]. The ABTS assay was performed using a procedure described by Miguel et al. [55]. The reducing power method was carried out according to method described by Oyaizu [56]. EC_50_ (mg/mL), which means a sample concentration that is able to scavenge 50% of a radical, was used to express the activity of the samples.

### 3.4. Antioxidant Potential of Propolis Samples

To determine the total phenolic content and flavone/flavonol content, the method described by Daraghmeh and Imtara [8] was adapted and used. This method has been widely used for determination of the antioxidant potential of propolis samples. The result of total phenolics was expressed as the mg gallic acid equivalent per gram of propolis (mg GAE/g), while the results of flavone and flavonol content were expressed as the mg quercetin equivalent per gram of propolis (mg QE/g). In addition, the estimated total antioxidant capacity (TAC) of samples was determined by the method described by Prieto et al. [57], the results of this method were expressed as mg ascorbic acid equivalent per gram of propolis (mg AAE/g).

### 3.5. HPLC Analysis

#### 3.5.1. Sample Preparation

About 100 mg of each sample was weighed, to which 10 mL of ethanol (70%) was added. After that, they were sonicated for 30 min at 50 °C. Then, the mixture was cooled at room temperature. The mixture was combined in a volumetric flask. Then, 10 mL of 70% ethanol was added and centrifuged at 3500 rpm (3403.45× *g*), and the supernatant was filtered and passed through a 0.45 µm syringe filter. Analysis was carried out by high-performance liquid chromatography.

#### 3.5.2. Method Development

The HPLC analysis was carried out using a Chromaster (Hitachi High Technologies, Schaumburg, IL, USA) HPLC system consisting of an auto sampler (5260), pump (5160), column oven (5310), and PDA detector (5430). The system was fitted with an ACE 5 C18 column (250 × 4.6 mm i.d., 5 μm) and a security guard cartridge. The following were the linear gradients of mobile phase methanol (solvent A) and 0.1% formic acid (solvent B) at a flow rate of 1 mL/min: 65% B, 0 min; 50% B, 8 min; 40% B, 15 min; 35% B, 25 min; 20% B, 40 min; 10% B, 60 min, and 10% B, 70 min. A 10 min equilibration time was used between runs. The chromatogram was monitored at 290 nm.

#### 3.5.3. Calibration Curve of Standards

The following standard references were used in this study: quercetin, *p*-coumaric acid, gallic acid, chlorogenic acid, ferulic acid, caffeic acid, cinnamic acid, rutin, naringenin, pinocembrin, chrysin, CAPE, and galangin. A series of dilutions were prepared from the stock solution (10 µg/mL) for each standard and then injected in the HPLC column to generate the calibration curves.

#### 3.5.4. Method Validation

Analytical validation followed the recommendations of the International Conference on Harmonization guidelines [58]. The bioanalytical method was validated in terms of the specificity, linearity, selectivity, precision, limit of detection, limit of quantitation, accuracy, interference (PDA detector), and robustness. 

The interference in the analysis due to the PDA detector, as more than one substance was quantified in this method, was analyzed to prioritize a wavelength at which molecules absorb proportionally, comparing the spectra of different molecules, and equivalent regions in the spectra were selected for the analysis.

Precision was determined as the intra-day and inter-day variation of results from analysis of three different concentrations of standard solutions. Intra-day and Inter-day precision were determined by triplicate analysis of each solution on the same day and the next day, respectively. The relative standard deviations (RSDs) of retention time (Rt) and AUC of standards were calculated as measures of precision, repeatability, and stability.

To determine the limits of detection (LOD) and quantification (LOQ), standard solutions were further diluted in methanol. LOD and LOQ were defined as the amounts for which signal to-noise (S/N) ratios were 3 and 10, respectively.

The accuracy of the method was determined by application of the standard addition method [59,60]. Accurately known amounts of the standards were added to 1 mL of pre-analyzed propolis sample and then analyzed in triplicate as described above. The total amount of each compound was calculated from the corresponding calibration plot, and the recovery of each compound was calculated by use of the following equation:

Recovery (%) = (amount found − amount contained)/amount added × 100(1)

### 3.6. Bacterial Strain and Inoculum Standardization

Gram-negative and Gram-positive bacterial strains were used for antibacterial activity studies. Propolis samples were tested against six bacterial strains including *E. coli* BLSE (ATB:87), *E. coli* (ATB:57), *E. coli* (ATB:97), and *Pseudomonas aeruginosa*, *Streptococcus faecalis*, and *Staphylococcus aureus*. All bacteria strains were provided by the Laboratory of Microbiology, Faculty of Medicine and Pharmacy, and Hassan II University Hospital, Fez. Table 6 shows the antibiotics applied for each strain studied.

### 3.7. Agar Well Diffusion (AWD) Assay

The antimicrobial activity of propolis samples was evaluated in triplicate according to the procedure described by Kirby-Bauer [61], with slight modifications. The antimicrobial screening was performed by using Mueller–Hinton agar (MHA). The agar plate surface is inoculated by physiological inoculum (108 cfu·mL^−1^). The bacterial suspension was prepared according to the method explained previously [62]. Then, the paper discs (Whatman, 6 mm) were placed on the surface of the pre-inoculated agar and impregnated with 10 μL propolis samples (stock solution: 100 mg/mL). The inoculated plates were incubated at 37 °C for 24 h. The diameter of the inhibition zone was measured in mm.

### 3.8. Determination of the Minimum Inhibitory Concentration (MIC) and Minimal Bactericidal Concentration (MBC)

A microdilution test in microplates (96-well plates) was used to determine the MICs of propolis samples according to NCCLS standards [63]. Serial hydro-ethanol (70%) dilution of each sample was prepared in sterile tubes with concentration ranging from 100 to 0.20 mg/mL. Then, 10 µL of each concentration was added into each well containing 170 µL of Mueller–Hinton broth and 20 µL of the bacterial suspension (5 × 105 CFU/mL). The plates were incubated at 37 °C for 20 h. After that, triphenyl tetrazolium chloride (40 µL) was added to each well. The MIC results were observed by the disappearance of the red color of TTC, and it is defined as the lowest concentration that prevented the red color [61].

Minimal bactericidal concentration (MBC) of propolis samples was determined according to method described by Rand et al. [64], and it is defined as the lowest concentration of the propolis that completely killed 99.9% of the inoculated bacteria.

### 3.9. Statistical Analysis

Biological activities of propolis samples were completed in triplicates, and the data were reported as mean ± SD. Statistical analysis were achieved by Pearson correlation coefficient (r) at a significance level of 99% (*p* < 0.01). The data pre-processing and the PCA were accomplished using MultBiplot64 running in MATLAB R2017a. The comparisons between the samples were performed using ANOVA through the SPSS 23 software and using the Tukey post hoc test at *p* < 0.05.

## 4. Conclusions

For the first time, the phenolic compound analysis of Palestinian propolis is studied and the results show that the samples from Palestine are rich in cinnamic acid component compared to the Moroccan propolis. Moreover, sample P4 from Palestine has important antioxidant and antibacterial activities. Our finding requires more detailed studies of the phenolic compounds on a large number of Palestinian propolis, through which it can be judged whether cinnamic acid can be used as an indicator of the quality of Palestinian propolis.

## Figures and Tables

**Table 1:** Moisture, ash, pH, wax, balsam, and resin contents of propolis samples.

Scheme	Wax (%)	Resin (%)	Balsam (%)	Moisture (%)	Ash (%)	pH
P1	26.09 ± 1.92 ^c^	56.33 ± 1.03 ^c^	1.02 ± 0.02 ^b^	2.05 ± 0.01 ^b^	3.42 ± 0.01 ^b^	5.2 ± 0.01 ^a^
P2	20.31 ± 1.03 ^d^	59.01 ± 0.12 ^a^	1.11 ± 0.01 ^ab^	1.01 ± 0.01 ^c^	4.83 ± 0.01 ^a^	5.1 ± 0.11 ^a^
P3	43.12 ± 1.23 ^a^	54.14 ± 0.19 ^d^	0.76 ± 0.01 ^c^	2.07 ± 0.02 ^b^	2.53 ± 0.02 ^c^	4.5 ± 0.2 ^bc^
P4	13.39 ± 1.08 ^e^	57.76 ± 0.08 ^b^	1.3 ± 0.03 ^a^	1.03 ± 0.01 ^c^	5.11 ± 0.01 ^a^	4.8 ± 0.2 ^ab^
P5	29.86 ± 1.02 ^b^	45.25 ± 0.13 ^e^	0.69 ± 0.01 ^c^	2.79 ± 0.09 ^a^	1.67 ± 0.02 ^d^	4.2 ± 0.1 ^c^

Values in the same column followed by the same letter are not significantly different according to Tukey’s multiple range tests (*p* < 0.05).

**Table 2:** The regression equations with R^2^ value, linearity, LOD, LOQ, precision, stability, repeatability, and accuracy.

Analyte	Equation of Calibration Curve	Determination Coefficient (R^2^)	Linear Range (μg/mL)	LOD (μg/mL)	LOQ (μg/mL)	Precision RSD (%), *n* = 3		Stability RSD (%)	Repeatability RSD (%)	Accuracy	
						Intra-Day AUC	Inter-Day AUC			Mean % Recovery	Mean RSD (%)
Caffeic acid	y = 577,684x + 504.35	0.9801	0.05–0.50	0.017	0.05	1.74	1.58	1.1	1.3	100.48	1.25
*p*-Coumaric acid	y = 2 × 10^6^x + 98.8	0.9999	0.01–0.05	0.003	0.01	1.92	1.85	1.5	0.5	100.37	1.69
Ferulic acid	y = 730,425x + 8016.8	0.9811	0.001–0.05	0.0003	0.001	2.23	2.10	0.8	1.0	100.57	1.97
Quercetin	y = 1 × 10^6^x − 14,970	0.9651	0.01–0.30	0.003	0.01	1.66	1.52	0.9	1.6	100.25	1.01
Cinnamic acid	y = 1 × 10^6^x − 176.2	0.9997	0.01–0.30	0.003	0.01	4.31	4.19	0.8	0.7	100.60	1.20
Naringenin	y = 9 × 10^6^x − 12,307	0.9996	0.01–0.10	0.003	0.01	6.50	6.37	1.0	0.8	100.39	1.10
Pinocembrine	y = 1 × 10^7^x − 1492.3	0.9998	0.01–1.00	0.003	0.01	5.19	4.99	1.2	1.3	100.51	1.65
Chrysin	y = 8 × 10^6^x − 1982.3	0.9999	0.005–0.05	0.0016	0.005	3.83	3.72	0.7	1.9	100.59	1.43
Galangin	y = 4 × 10^6^x + 6009.3	0.9947	0.01–0.30	0.003	0.01	4.55	4.47	1.2	1.4	100.44	1.23

LOD: limit of detection; LOQ: limit of quantification; RSD: relative standard deviation; AUC: area under curve.

**Table 3:** The levels of nine marker compounds obtained from the analysis of Moroccan and Palestinian propolis.

Amounts of Marker Compounds (µg/g)					
Standard	P1	P2	P3	P4	P5
Caffeic acid	431	2152	108	572	nd
*p*-Coumaric acid	1110	2258	157	2479	nd
Ferulic acid	75.04	1829	89.18	1867	nd
Quercetin	nd	nd	1591	nd	nd
Cinnamic acid	820	nd	nd	21,513	2901
Naringenin	599	8988	583	2805	nd
Pinocembrine	8721	352,001	12,423	25,445	nd
Chrysin	542	30,062	1384	598	nd
Galangin	234	25,014	643	5856	nd

nd: not determined.

**Table 4:** Total phenolic content, flavone and flavonol content, TAC, and antioxidant activities of propolis samples.

Samples	Total Phenolic Content (mg GAE/g)	Flavone and Flavonol Content (mg QE/g)	TAC (mg AAE/g)	DPPH EC_50_ (mg/mL)	ABTS EC_50_ (mg/mL)	Reducing Power EC_50_ (mg/mL)
P1	122 ± 0.81 ^c^	76.52 ± 0.41 ^d^	83.34 ± 1.20 ^a^	0.08 ± 0.02 ^b^	0.02 ± 0.001 ^d^	0.06 ± 0.002 ^c^
P2	148 ± 1.31 ^a^	118 ± 1.92 ^a^	90.87 ± 2.91 ^a^	0.02 ± 0.02 ^c^	0.05 ± 0.001 ^a^	0.04 ± 0.001 ^e^
P3	115 ± 1.42 ^c^	98.21 ± 0. 41 ^c^	67.82 ± 3.46 ^b^	0.07 ± 0.01 ^bc^	0.04 ± 0.01 ^b^	0.07 ± 0.006 ^b^
P4	136 ± 1.73 ^b^	107 ± 0.98 ^b^	87.67 ± 1.92 ^a^	0.04 ± 0.001 ^bc^	0.03 ± 0.02 ^c^	0.05 ± 0.006 ^d^
P5	74.71 ± 0.89 ^d^	26.97 ± 2.44 ^e^	48.01 ± 0.51 ^c^	0.14 ± 0.01 ^a^	0.04 ± 0.01 ^b^	0.09 ± 0.003 ^a^

Values in the same column followed by the same letter are not significantly different according to Tukey’s multiple range tests (*p* < 0.05).

**Table 5:** Results of the antibacterial activity of propolis samples.

Samples	Tests	*E. coli* BLSE (ATB:87) BGN	*E. coli* (ATB:57) B6N	*E. coli* (ATB:97) BGM	*Pseudomonas* *aeruginosa*	*Streptococcus* *faecalis*	*Staphylococcus* *aureus*
P1	DI (mm)	17.23 ± 1.21 ^ab^	13.6 ± 0.5 ^c^	16.3 ± 1.26 ^bc^	10.3 ± 0.6 ^bc^	21.33 ± 1.57 ^c^	23 ± 1.12 ^b^
	MIC (mg/mL)	0.625	2.50	1.25	1.25	0.625	0.31
	MBC (mg/mL)	1.25	>5	2.50	>5	1.25	0.31
P2	DI (mm)	21.33 ± 1.52 ^a^	19.76 ± 0.40 ^b^	20.66 ± 1.57 ^a^	15.54 ± 1.1 ^ab^	32.5 ± 1.02 ^a^	31.2 ± 1.73 ^a^
	MIC (mg/mL)	0.31	0.31	0.31	0.625	0.09	0.09
	MBC (mg/mL)	0.31	0.625	0.31	1.25	0.09	0.09
P3	DI (mm)	13.31 ± 1.57 ^b^	-	15.22 ± 0.56 ^c^	9.66 ± 0.6 ^c^	27 ± 1.18 ^b^	25 ± 0.57 ^b^
	MIC (mg/mL)	1.25	5	1.25	>5	0.625	1.25
	MBC (mg/mL)	>5	>5	2.50	>5	0.625	1.25
P4	DI (mm)	18.71± 1.73 ^ab^	21.8 ± 0.35 ^a^	19.76 ± 0.40 ^ab^	20.33 ± 0.57 ^a^	22.8 ± 1.25 ^bc^	27.66 ± 0.57 ^ab^
	MIC (mg/mL)	0.31	0.31	0.625	0.625	0.17	0.17
	MBC (mg/mL)	0.31	0.31	0.625	1.25	0.17	0.17
P5	DI (mm)	14.33 ± 1.15 ^b^	14.6 ± 0.5 ^c^	13.53 ± 0.89 ^c^	-	18 ± 0.57 ^c^	13 ± 1 ^c^
	MIC (mg/mL)	1.25	2.50	2.50	-	0.625	1.25
	MBC (mg/mL)	>5	>5	>5	-	0.625	2.50
Eth70%	DI (mm)	-	-	-	-	-	-
	MIC (mg/mL)						
	MBC (mg/mL)						

Eth70%: ethanol 70%; DI: Diameter of Inhibition; MIC: Minimum Inhibitory Concentration; MBC: Minimal Bactericidal Concentration; -: not determined. Values in the same column followed by the same letter are not significantly different according to Tukey’s multiple range tests (*p* < 0.05).

**Table 6:** List of antibiotic resistance applied to the studied bacteria.

Bacterial Strains	Antibiotic Resistance
*E. coli* BLSE (ATB:87) BGN	CXM, CRO, CEC, AMX, CAZ, CTX, KF, and CIP
*E. coli* (ATB:57) B6N	CXM, AMX, CTX, KF, SXT, and CIP
*E. coli* (ATB:97) BGM	AMX
*Pseudomonas aeruginosa*	SXT and AMC
*Streptococcus faecalis*	SXT, TE, VA, E, P, and OX
*Staphylococcus aureus*	VA

CXM: cefuroxime; CRO: ceftriaxone; CEC: cefaclor; AMX: amoxicillin; CAZ: ceftazidime; CTX: cefotaxime KF: cephalothin; CIP: ciprofloxacin; SXT: trimethoprim-sulfamethoxazole; AMC: amoxicillin/clavulanate; TE: tetracycline; VA: vancomycin; E: erythromycin; P: penicillin; OX: oxacillin.

**Table 7:** Pearson correlation coefficients between the bioactive compounds and the assessed activities of propolis samples.

	Total Phenolic	Flavone and Flavonol	TAC	DPPH	ABTS	Reducing Power	DI *E. coli* 87	DI *E. coli* 57	DI *E. coli* 97	DI *P. aeruginosa*	DI *S.* *faecalis*	DI *S.* *aureus*	MIC *E. coli* 87	MIC *E. coli* 57	MIC *E. coli* 97	MIC *P. aeruginosa*	MIC *S.* *faecalis*	MIC *S.* *aureus*
Total phenolic	1	0.953 *	0.968**	−0.984 **	−0.068	−0.994 **	0.793	0.342	0.923 *	0.917 *	0.763	0.982 **	−0.841	−0.507	−0.995 **	0.543	−0.762	−0.827
Flavone and flavonol	0.953 *	1	0.862	−0.978 **	0.008	−0.914 *	0.609	0.121	0.839	0.897 *	0.826	0.990 **	−0.667	−0.312	−0.970 **	0.304	−0.698	−0.629
TAC	0.968 **	0.862	1	−0.915 *	−0.257	−0.984 **	0.825	0.448	0.900 *	0.909 *	0.596	0.908 *	−0.910 *	−0.575	−0.947 *	0.725	−0.709	−0.918 *
Caffeic acid	0.753	0.644	0.687	−0.760	0.480	−0.776	0.887 *	0.517	0.822	0.534	0.807	0.727	−0.723	−0.660	−0.745	0.335	−0.807	−0.708
*p*-Coumaric acid	0.850	0.724	0.883 *	−0.836	−0.085	−0.870	0.921 *	0.765	0.968 **	0.898 *	0.457	0.778	−0.978 **	−0.863	−0.853	0.560	−0.915 *	−0.935*
Ferulic acid	0.768	0.713	0.725	−0.815	0.211	−0.762	0.848	0.716	0.947 *	0.834	0.551	0.746	−0.850	−0.839	−0.801	0.230	−0.992 **	−0.761

** The correlation is significant at the 0.01 level; * the correlation is significant at the 0.05 level; DI: diameter of inhibition.

**Table 8:** Region and Country of propolis samples collected with predominant vegetation.

Codes	Region and Country	Predominant Vegetation	Year of Harvest
P1	Fez Region, Morocco	*Pistacia, Olea, Pinus, Quercus, Juniperus, Rosmarinus, Cistus* and *Lavandula*	2017
P2	Sefrou region, Morocco	*Pistacia, Olea, Pinus, Quercus, Juniperus, Rosmarinus, Cistus* and *Lavandula*	2017
P3	Boulemane Region, Morocco	*Ceratonia, Silybum, Thymus, Juniperus, Rosmarinus*	2017
P4	Jenin, Palestine	*Olea, Citrus, Prunus*	2017
P5	Ramallah, Palestine	*Olea, Citrus, Prunus*	2017

## Data Availability

The data presented in this study are available on request from the corresponding author.

## Sample Availability

Samples are available from the authors upon reasonable request.
